# Prevalence and Clinical Correlation of Decayed, Missing, and Filled Teeth in Elderly Inpatients With Schizophrenia

**DOI:** 10.3389/fpsyt.2021.728971

**Published:** 2021-09-14

**Authors:** Mi Yang, Qiwen Li, Chijun Deng, Gang Yao, Xue Bai, Xi Tan, Xiangyang Zhang

**Affiliations:** ^1^The Fourth People's Hospital of Chengdu, Chengdu, China; ^2^MOE Key Laboratory for Neuroinformation, The Clinical Hospital of Chengdu Brain Science Institute, University of Electronic Science and Technology of China, Chengdu, China; ^3^MOE Key Laboratory for Neuroinformation, High-Field Magnetic Resonance Brain Imaging Key Laboratory of Sichuan, University of Electronic Science and Technology of China, Chengdu, China; ^4^CAS Key Laboratory of Mental Health, Institute of Psychology, Chinese Academy of Sciences, Beijing, China; ^5^Department of Psychology, University of Chinese Academy of Sciences, Beijing, China

**Keywords:** oral health, DMFT, cognitive impairment, schizophrenia, elderly inpatients

## Abstract

**Introduction:** Schizophrenia is a mental disease with a profound impact on human health. Patients with schizophrenia have poor oral hygiene, increasing their risk of systemic diseases, such as respiratory infections, and declining their quality of life. Therefore, this study aims to assess the oral health status of inpatients with schizophrenia, analyze its related factors, and thus provide scientific evidence for further exploration of corresponding control strategies.

**Methods:** A total of 425 inpatients older than 50 years with a diagnosis of schizophrenia from two psychiatric hospitals (mean age 58.49 ± 5.72 years) were enrolled. The demographic data of the patients were checked on admission. Two independent dentists examined caries, missing teeth, and fillings. Mini-Mental State Examination (MMSE) and Global Deterioration Scale were performed as cognitive tests. Positive and Negative Syndrome Scale and Repeatable Battery for the Assessment of Neuropsychological Status rating scale were used to determine their mental status.

**Results:** The average decayed, missing, and filled teeth index was 12.99 ± 8.86. Linear regression analysis showed that the decayed, missing, and filled teeth index had a significantly positive relationship with age (*p* < 0.001) and smoking (*p* < 0.001) and a negative relationship with MMSE (*p* = 0.029). The missing teeth index had a positive relationship with age (*p* < 0.001), smoking (*p* < 0.001), and Global Deterioration Scale (*p* = 0.014) and a negative relationship with MMSE (*p* = 0.004).

**Conclusion:** The oral health of elderly patients with schizophrenia is poor, which may be related to the cognitive level of patients and affect their quality of life. The focus should be provided to the oral care of patients with schizophrenia, and investment in their specialized oral treatment should be increased.

## Introduction

The incidence of mental disorders has rapidly increased worldwide, inducing a series of severe family, social, and cultural problems and affecting the development of the economy. Schizophrenia is a complex mental disease with a profound impact on human health; it is a chronic mental disorder affecting 20 million people globally, as revealed by the “Global Burden of Illness, Injury, and Risk Factors study” in 2017 (1). In China, the prevalence of schizophrenia was estimated to range from 0.4 to 0.5% (2), with the elderly accounting for ~30.0% (3).

Elderly patients with schizophrenia have mental disease *per se* and oral hygiene and health problems resulting from frequent medication and inadequate oral cleaning care (4, 5). Elderly inpatients with schizophrenia present with special diverse disease symptoms, volatile emotional behavior, and decreased intellectual, cognitive level (6). Incomplete oral cleaning and insufficient hygienic management cause poor oral hygiene and significantly increase the incidence of oral diseases. The decayed, missing, and filled teeth (DMFT) index of hospitalized psychiatric patients increases with age (7). Some studies suggested that the oral health of hospitalized elderly patients with mental illness is impaired (8). Oral health problems undoubtedly increase the risk of lung infections, such as aspiration pneumonia (9). A large number of studies have demonstrated that the incidence of nosocomial respiratory tract infection is considerably higher in elderly patients in psychiatric hospitals than in other parts of the human body (10–12), and this incidence is even higher than that in general hospitals.

Recent studies have focused on the interaction between poor oral health and cognitive impairment (13). The incidence of oral health problems in elderly patients with cognitive impairment has increased significantly. Oral pathogenic bacteria, such as *Porphyromonas gingivalis*, and inflammatory factors in the mouth can affect protein regulatory channels and pass through the blood–brain barrier, further aggravating cognitive impairment. Several previous studies investigated the Mini-Mental State Examination (MMSE) scale and the missing teeth (MT) index and concluded that tooth loss is a related factor of cognitive impairment or its deterioration (14–18). Some other studies assessed the effect of positive and negative symptoms on the DMFT index in patients with schizophrenia. They found that the Positive And Negative Syndrome Scale (PANSS) negative subscale (PANSS-N) score is positively correlated with the oral health variables and that the PANSS positive subscale (PANSS-P) score is negatively correlated with MT (19). However, few studies have investigated the relationship between the DMFT index and cognitive impairment. In particular, reports on the correlation between the oral health status and cognitive level using the DMFT index in elderly patients with mental illness are lacking.

Therefore, this study analyzed the oral health status and related factors of elderly patients with schizophrenia from two large-scale psychiatric hospitals. Specifically, we assessed teeth status in patients with schizophrenia and addressed the relationship with cognitive and psychiatric status. Oral health management strategies were proposed to effectively reduce the risk of lower respiratory tract infections and improve the comprehensive medical services of psychiatric hospitals and the quality of life of elderly patients with schizophrenia.

## Methods and Materials

### Sample Selection and Population

We used a cross-sectional study in two psychiatric hospitals on elderly patients with schizophrenia. From 2018 to 2019, 425 hospitalized patients with schizophrenia diagnosed by the International Classification of Diseases, 10th Revision, included 265 from the Guangdong Hospital and 160 from the Wuhan Hospital (Table 1). All long-term patients with schizophrenia aged 50 years and older were included in this study. Patients with major basic diseases and acute onset of schizophrenia were excluded. Demographic data included sex, age, education level, marriage, smoking, height, weight, diabetes, hypertension, and use of psychiatric medications. Body mass index (BMI) was calculated from height and weight (BMI = kg/m^2^). In accordance with the Chinese reference standard, they were classified as normal (18.5 ≤ BMI < 24), lean (BMI < 18.5), overweight (24 ≤ BMI < 28), and obese (BMI ≥ 28). The DMFT index was used to assess oral health, whereas cognitive and mental statuses were measured.

**Table 1:** DMFT scores of elderly schizophrenia inpatients in different hospitals.

**Hospitals**	**Number**	**Percentage (%)**	**DMFT**	**SD**	***t***	***p*** **-value**
Guangzhou	265	62.4	14.25	8.93	3.855	<0.001
Wuhan	160	37.6	10.89	8.37		
total	425	100.0	12.99	8.86		

The Ethics Committee of the Institute of Psychology, Chinese Academy of Sciences, approved this study. Ethical approval was conducted in accordance with the latest version of the Helsinki Declaration (lines 96–98). Written informed consent was obtained from all participants.

### Dental Examination

Two dentists had been trained before this study. Consistency was assessed by Kappa calibration, and the Kappa value was >0.8. The decayed teeth (DT: number of teeth or surfaces with caries in the mouth), MT (number of teeth lost in the mouth due to caries; dental decay and periodontal disease can no longer be distinguished at age 45 years and older), filled teeth (FT: number of teeth or surfaces that have been filled for caries), and DMFT indices were detected under artificial light in a dental chair.

### Measurement of Cognition and Mental States

Cognitive function was evaluated with the MMSE and Global Deterioration Scale (GDSRANK) (20). MMSE is mainly concerned with the ability of orientation, registration, attention, calculation, recall, and language (21). The MMSE score ranges from 0 to 30, and a lower score indicates poorer cognitive ability. Each question has three possible answers: true, false, and unanswerable. We counted the unanswerable as incorrect answers (22). GDSRANK assesses dementia severity over seven grades (23): absence of cognitive changes (first grade), very mild cognitive decline (second grade), mild cognitive decline (third grade), moderate cognitive decline (fourth grade), moderately severe cognitive decline (fifth grade), severe cognitive decline (sixth grade), and very severe cognitive decline (seventh grade). The score was derived from the interview of patients and caregivers.

Mental status was evaluated with PANSS (24) and Repeatable Battery for the Assessment of Neuropsychological Status rating scale (RBANS) (25). Scores of PANSS-P, PANSS-N, PANSS general psychopathology (PANSS-G), and total were applied separately. The RBANS consists of 12 task tests assessing five aspects of neuropsychological function, namely, immediate memory, visuospatial structure, language, attention, and delayed memory.

### Statistical Analysis

IBM SPSS 24.0 was used to analyze the data with *t*-tests. Mann–Whitney *U*-test or Kruskal–Wallis *H*-test was used for variables with non-normality or homogeneity of variance. The Kolmogorov–Smirnov test was used to test normality. Counting variables were analyzed by the chi-squared test or *Fisher's* exact probability test. The relationship between metrological/classification data and DMFT was analyzed using the Pearson or Spearman correlation method. The risk factor analysis for DMFT and MT in elderly hospitalized schizophrenics was performed through linear regression. The statistical significance level was set at 0.05.

## Results

The mean DMFT score of the 425 patients was 12.99 ± 8.86, 14.25 ± 8.93 for patients in Guangzhou and 10.89 ± 8.37 for patients in Wuhan (Table 1). Patients in the two hospitals showed a significant difference (*t* = 3.855, *p* < 0.001). The total mean DT value was 4.61 ± 5.03, the MT value was 7.82 ± 8.39, and the FT value was 0.56 ± 2.34. The total caries rate, missing rate, and filling rate were 83.1, 83.3, and 14.6%, respectively.

### Demography and Decayed, Missing, and Filled Teeth Index

The Kolmogorov–Smirnov test showed that the DMFT and MT indices were not normal (*p* = 0.001). Table 2 presents the demographic characteristics of elderly inpatients with schizophrenia. Sex (*p* = 0.001), age (*p* < 0.001), education level (*p* = 0.038), and smoking status (*p* < 0.001) significantly influenced the DMFT scores of the patients. High DMFT scores were significantly associated with age and smoking. Female participants and those with high education levels showed significantly lower DMFT scores than their counterparts.

**Table 2:** DMFT scores of elderly schizophrenia inpatients according to demographic.

**Variable**		**Number**	**Percentage (%)**	**DMFT**		***U*** **or** ***H***	***p*** **-value**
				**Mean**	**SD**		
Gender	Male	295	69.4	13.90	9.07	10.532	0.001
	Female	130	30.6	10.91	8.03		
Age	50–54	123	28.9	8.62	7.51	55.329	<0.001
	55–59	121	28.5	13.17	8.84		
	60–64	98	23.1	15.01	8.55		
	≥65	83	19.5	16.81	8.46		
Education	Primary and below	67	15.8	14.55	9.40	8.408	0.038
	Junior high	166	39.0	12.95	9.00		
	Senior high	152	35.8	13.29	8.68		
	University and above	40	9.4	9.38	7.23		
Marital status	Single	233	54.8	13.59	9.09	4.706	0.095
	Married	112	26.4	13.01	8.44		
	Others	80	18.8	11.20	8.63		
Smoking	No	210	49.4	10.64	8.44	37.322	<0.001
	Used to	62	14.6	13.00	7.69		
	Smoking now	153	36.0	16.20	8.91		
BMI	Lean	28	6.6	12.93	9.61	7.531	0.057
	Normal	195	45.9	14.18	9.00		
	Overweight	130	30.6	12.14	8.14		
	Obesity	72	16.9	11.29	9.15		
Diabetes	No	325	76.5	12.93	8.87	0.063	0.803
	Yes	100	23.5	13.18	8.87		
Hypertension	No	315	74.1	12.77	8.87	0.691	0.406
	Yes	110	25.9	13.59	9.10		
Number of Medication	1	242	56.9	13.96	9.23	6.006	0.050
	2	163	38.4	11.91	8.32		
	3	20	4.7	10.00	7.07		

### Cognitive and Mental Statuses Against the Decayed, Missing, and Filled Teeth Index

Table 3 shows that the DMFT index was significantly correlated with age (*r* = 0.33, *p* < 0.001), total MMSE score (*r* = −0.17, *p* = 0.001), GDSRANK score (*r* = 0.13, *p* = 0.006), PANSS-N score (*r* = 0.11, *p* = 0.024), and PANSS-G score (*r* = 0.11, *p* = 0.031) but not with PANSS-P score (*r* = −0.03, *p* = 0.607), PANSS total score (*r* = 0.09, *p* = 0.06), or RBANS score (*r* = −0.11, *p* = 0.071).

**Table 3:** Correlations of DMFT and MT with cognition and mental state.

**Variable**	**DMFT**		**MT**	
	***r***	***p***	***r***	***p***
Age	0.33	<0.001	0.43	<0.001
Total MMSE	−0.17	0.001	−0.21	<0.001
GDSRANK	0.13	0.006	0.21	<0.001
PANSS-N	0.11	0.024	0.12	0.014
PANSS-P	−0.03	0.607	−0.00	0.120
PANSS-G	0.11	0.031	0.12	0.016
PANSS total	0.09	0.060	0.11	0.025
RBANS	−0.11	0.071	−0.13	0.031

### Cognitive and Mental Statuses Against Missing Teeth Index

Table 3 shows that the MT index was significantly correlated with age (*r* = 0.43, *p* < 0.001), total MMSE score (*r* = −0.21, *p* < 0.001), GDSRANK score (*r* = 0.21, *p* < 0.001), PANSS-N score (*r* = 0.12, *p* = 0.014), PANSS-G score (*r* = 0.12, *p* = 0.016), PANSS total score (*r* = 0.11, *p* = 0.025), and RBANS score (*r* = −0.13, *p* = 0.031) but not with PANSS-P score (*r* = −0.00, *p* = 0.120).

### Linear Regression Model of Decayed, Missing, and Filled Teeth

As shown in Table 4, age (β = 0.469, *p* < 0.001), smoking (β = 2.616, *p* < 0.001), and MMSE score (β = −0.128, *p* = 0.029) are risk factors for the DMFT index. The DMFT index increased with age and smoking and decreased with MMSE score ascending. The association between MMSE and DMFT scores remained significant after controlling for age and smoking (*p* < 0.05).

**Table 4:** Linear regression model of DMFT in elderly schizophrenia inpatients.

**DMFT**	**β**	***t***	**95.0% CI**		***p*** **-value**
			**Lower**	**Upper**	
Age	0.469	6.802	0.333	0.604	<0.001
Smoking	2.616	6.160	1.781	3.450	<0.001
Total MMSE	−0.128	−2.195	−0.242	−0.013	0.029

### Linear Regression Model of Missing Teeth

As shown in Table 5, age (β = 0.595, *p* < 0,001), smoking (β = 1.157, *p* < 0.001), and GDSRANK score (β = 0.661, *p* = 0.014) are positive risk factors for MT index, whereas total MMSE score (β = −0.156, *p* = 0.004) is a negative risk factor of MT score. After controlling for age and smoking, total MMSE score and GDSRANK score remained significantly associated with the MT index (*p* < 0.05).

**Table 5:** Linear regression model of MT in elderly schizophrenia inpatients.

**MT**	**β**	***t***	**95.0% CI**		***p-value***
			**Lower**	**Upper**	
Age	0.595	9.173	0.467	0.722	<0.001
Smoking	1.157	2.921	0.379	1.936	<0.001
Total MMSE	−0.156	−2.877	−0.262	−0.049	0.004
GDSRANK	0.661	2.467	0.134	1.187	0.014

## Discussion

Poor oral hygiene in elderly psychiatric inpatients may aggravate cognitive impairment and increase the risk of aspiration pneumonia infection, resulting in a high incidence of hospital infections. The oral health of these patients can be improved by implementing professional oral treatment or nursing during hospitalization (26). Improved oral hygiene can reduce the incidence of respiratory diseases (27) and decrease the incidence of nosocomial infections. The regained mastication function can also improve the cognitive performance of elderly patients (26). Therefore, the oral health of elderly inpatients with schizophrenia needs special attention.

A large number of previous studies examined the relationship between the MT index and cognitive impairment of the elderly in the community or the general population (15, 28, 29), but few analyzed the relationship between the DMFT index and cognitive level. Studies on patients with schizophrenia focused on their oral conditions. Therefore, we investigated the oral health status of elderly inpatients with schizophrenia on the basis of the DMFT index and examined the correlation between DMFT and the cognition or clinical symptoms of mental illness.

### Descriptive Analysis of Oral Health

The prevalence rates of dental caries and tooth loss in elderly patients with schizophrenia were 83.1 and 83.3%, respectively, consistent with the previously reported caries rate of ~80.0% (30, 31) and MT rate of 81.4% (7) in psychiatric patients. Poor oral hygiene indicates a serious lack of oral health care.

Several published studies to date analyzed the oral health of inpatients with mental disorders by using the mean DMFT score, such as 20.6, 15.8, and 16.6 (8, 32, 33), which were higher compared with the 12.99 obtained in the present study. Several possible reasons may explain these inconsistent results. First, ethnic and regional differences existed between studies. The two surveyed areas in China are relatively developed regions. Different countries show large differences in DMFT value (8, 32–34). Second, the present study included only patients with schizophrenia, whereas other studies included patients with other mental disorders, such as substance addiction, mania, bipolar disorder, and depression, which may cause even worse oral health. Third, sample sizes were different. The sample size in this study was relatively larger than those in the other studies (i.e., no more than 200 patients). Among the cases collected, the proportion of Guangzhou hospital was higher than that of Wuhan hospital, and the composition of age and sex was also different.

### Demographic Effects of Decayed, Missing, and Filled Teeth Values

The DMFT score of males was higher than that of females (*U* = 10.532, *p* = 0.001), consistent with most previous results (35, 36). This sex difference may relate to the poorer awareness of oral self-management (36) and smoking habits in male patients with schizophrenia. The DMFT score significantly increased with age (*H* = 55.329, *p* < 0.001). The effect of age on oral health was confirmed by a large number of studies (37). As age increases, the functions of chewing and swallowing in the elderly decline, and the incidence of gum atrophy and periodontal disease increases, directly affecting their oral health and nutritional status. The increased education level is significantly related to decreased DMFT (*H* = 8.408, *p* = 0.038), which is in line with the finding of Vano et al. (36). This result may be because people with higher education are more obedient to oral management and willing to require treatment for dental caries. Smoking is also a significant factor influencing dental caries and tooth loss (38, 39). A study on tobacco use and oral conditions showed that chewing tobacco and smoking are important risk factors for dental caries (40). Tobacco affects human gingival health and the morphology and moving ability of periodontal ligament fibroblasts, inducing the development and progression of periodontal disease (31), which can easily cause dental caries and tooth loss.

### Relationship Between Decayed, Missing, and Filled Teeth/Missing Teeth and Cognition

Previous studies often used the Montreal Cognitive Assessment (28) and MMSE (17, 18) to conduct the cognitive evaluation of patients. This study enrolled elderly schizophrenia patients with cognitive impairment. Therefore, we chose the MMSE scale, a relatively simple intelligence scale to quantify the cognitive level of patients. DMFT and MT were correlated with MMSE scores, and this correlation remained even after controlling for confounders, including age and smoking. In a Japanese cohort study on community-dwelling older adults, tooth loss was independently associated with the development of cognitive impairment within 4 years, confirming the hypothesis that tooth loss is a predictor or risk factor for cognitive decline (29). Another 13-year longitudinal study showed that tooth loss is associated with a cognitive decrease in older Chinese (15). Several cross-sectional studies demonstrated that DMFT relates to tooth loss and cognition (17, 18, 28). In addition, the nursing group used GDSRANK to assess neurodegeneration of the patients (23), and the results indicated that MT remained associated with GDSRANK score after controlling for age and smoking, whereas DMFT was not. Few studies used GDSRANK as a cognitive scale, but cross-sectional studies identified a weak association between cognitive impairment and MT (41). Therefore, we contend that MT plays an important role in cognitive impairment in elderly patients with schizophrenia.

Tooth loss in adults is mainly attributed to periodontal disease and dental caries (42). Therefore, tooth loss can be regarded as an indicator of severe inflammation caused by oral microorganisms. Periodontal disease caused by *P. gingivalis* may be associated with increased antibody levels (43). A possible reason may be that oral pathogenic bacteria and inflammatory factors enter the blood–brain barrier through influencing protein regulation channels, which further aggravate cognitive dysfunction. Noble et al. (43) showed that patients with high levels of antibodies to *P. gingivalis* have observably greater odds of impaired verbal memory and subtraction test performance than individuals with low levels.

Tooth loss can cause deficiency or decline of chewing function, resulting in changes in brain function (28). Chen et al. (44) found that active chewing can improve thinking ability and memory by promoting heart function and increasing the secretion of related hormones. Meanwhile, chewing can increase the secretion of saliva, and brain areas associated with salivary production are involved in memory and learning. Thus, the more teeth are missing, the worse chewing function is. This condition may indirectly affect cognitive function. Therefore, reconstruction of masticatory function may exert a positive effect on the prevention of cognitive decline in the elderly.

### Relationship Between Decayed, Missing, and Filled Teeth/Missing Teeth and the Mental State

Arnaiz et al. (19) showed that the PANSS-N score is associated with poor oral health, suggesting that symptomatology of schizophrenia is a risk factor for poor oral health. The present study showed that PANSS-N and PANSS-G scores were positively correlated with DMFT and MT, whereas PANSS total score was only positively correlated with MT. This correlation disappeared when controlling for age and smoking confounders. Negative symptoms, such as intellectual disability, lack of interest and emotion, and loss of life motivation, may lead to the reduced oral self-cleaning ability of patients with schizophrenia, unable to consciously maintain oral hygiene (45–48). However, elderly inpatients with schizophrenia are often accompanied by difficulties in self-management of life and need to complete daily oral cleaning under the care of others. Delusions and auditory hallucinations associated with positive symptoms may trigger impulsive behavior, resulting in a mixture of traumatic tooth loss factors (46). As a result, no relevance was found between the subscales of symptoms and DMFT or MT when the confounding age and smoking were eliminated.

In conclusion, long-term elderly inpatients with schizophrenia have cognitive impairments themselves. Their limited oral self-maintenance capacity, combined with the potential conflict between respecting patients' autonomy and providing good daily care (49), makes many effective oral care practices difficult to implement, further exacerbating the lack of oral health management in this population. This condition may not only aggravate the cognitive impairment of patients and affect the quality of life but also increase the risk of respiratory nosocomial infection and seriously hamper the improvement of the quality of medical services. Therefore, strengthening the specialized training of oral care skills should be strengthened, and the investment in professional oral treatments must be increased to help these patients obtain good oral health, quality of life, and self-esteem.

## Limitations

Oral health refers to the comprehensive condition of the oral cavity. In this study, only one dimension of the DMFT index was evaluated, and other oral function indexes such as chewing, swallowing, and speech were not included. In determining the mean of caries, an oral curved slice was not used to investigate the root caries alone. In addition, this study was a multicenter cross-sectional study. A regional difference in the dietary habits of patients may also lead to biased results.

## Data Availability Statement

The raw data supporting the conclusions of this article will be made available by the authors, without undue reservation.

## Ethics Statement

The studies involving human participants were reviewed and approved by the Ethics Committee of Institute of Psychology, Chinese Academy of Sciences. The patients/participants provided their written informed consent to participate in this study.

## Author Contributions

MY and QL searched and reviewed the literature, analyzed the data, and wrote the manuscript. CD and GY searched the literature and analyzed the data. XB and XT distributed questionnaires and collected data. XZ negotiated with the hospitals to conduct this study, collected data, and assisted in finding documents, issuing questionnaires, and analyzing the data. All authors contributed to the article and approved the submitted version.

## Funding

This study was supported by the National Natural Science Foundation of China (grant numbers 61806042 and 62073058); the Special Research Project for the novel coronavirus pneumonia funded by the Chengdu Science and Technology Bureau (grant number 2020-YF05-00171-SN); and the Science and Technology Department of Sichuan Province (grant number 2018sz0236).

## Conflict of Interest

The authors declare that the research was conducted in the absence of any commercial or financial relationships that could be construed as a potential conflict of interest.

## Publisher's Note

All claims expressed in this article are solely those of the authors and do not necessarily represent those of their affiliated organizations, or those of the publisher, the editors and the reviewers. Any product that may be evaluated in this article, or claim that may be made by its manufacturer, is not guaranteed or endorsed by the publisher.
